# Excited State Intramolecular Proton Transfer Dynamics of Derivatives of the Green Fluorescent Protein Chromophore

**DOI:** 10.3390/ijms24043448

**Published:** 2023-02-09

**Authors:** Junghwa Lee, Pyoungsik Shin, Pi-Tai Chou, Taiha Joo

**Affiliations:** 1 Department of Chemistry, Pohang University of Science and Technology (POSTECH), Pohang 37673, Republic of Korea; 2 Department of Chemistry, National Taiwan University, Taipei 10617, Taiwan, China

**Keywords:** excited state intramolecular proton transfer, time-resolved fluorescence, nuclear wave packets, GFP chromophore

## Abstract

Excited state intramolecular proton transfer (ESIPT) dynamics of the *o*-hydroxy analogs of the green fluorescent protein (GFP) chromophore have been investigated by time-resolved spectroscopies and theoretical calculations. These molecules comprise an excellent system to investigate the effect of electronic properties on the energetics and dynamics of ESIPT and to realize applications in photonics. Time-resolved fluorescence with high enough resolution was employed to record the dynamics and the nuclear wave packets in the excited product state exclusively in conjunction with quantum chemical methods. The ESIPT are ultrafast occurring in 30 fs for the compounds employed in this work. Although the ESIPT rates are not affected by the electronic properties of the substituents suggesting barrierless reaction, the energetics, their structures, subsequent dynamics following ESIPT, and possibly the product species are distinct. The results attest that fine tuning of the electronic properties of the compounds may modify the molecular dynamics of ESIPT and subsequent structural relaxation to achieve brighter emitters with broad tuning capabilities.

## 1. Introduction

Green fluorescent protein (GFP) has been exploited extensively in fluorescence microscopy of diverse biological systems, as its gene can be introduced and expressed in cells to provide strong and stable fluorescence [1,2,3]. The chromophore of GFP, 4-(4-hydroxy benzylidene)-1,2-dimethyl-1H-imidazol-5(4H)-one (*p*-HBDI), is formed from three intrinsic amino acid residues [4]. Upon electronic excitation of GFP, excited state intermolecular proton transfer occurs, which transforms the chromophore to the fluorescent anionic state.

The dynamics of the GFP chromophore in a protein matrix and solution have been studied extensively. The GFP chromophore is located in the central α-helix and the 11-stranded β-barrel [3], and the microscopic environment of the GFP chromophore strongly influences its photophysical properties. The chromophore shows low fluorescence quantum yield in solution compared with that in an intact protein environment [5,6,7,8]. Temperature dependence of the steady-state fluorescence spectrum of the chromophore in solution is different from that in a protein environment, showing less vibronic structure in solution at higher temperatures [9]. Analysis of internal conversion rates revealed a correlation to the conformational variance of *p*-HBDI, indicating more degrees of freedom in the nuclear coordinates of *p*-HBDI in solution [10,11,12]. These works assumed that the internal conversion is induced by a volume-conserving motion that is similar to the isomerization with a “hula twist” [13].

**Scheme 1:**
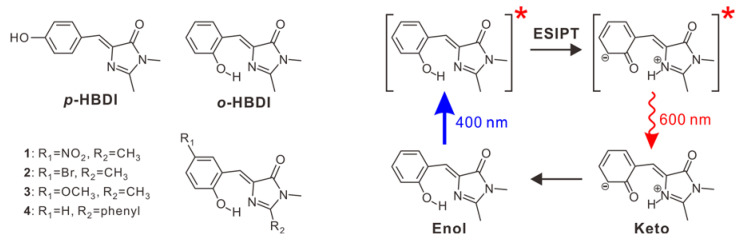
Structures of *p*-HBDI, *o*-HBDI, and derivatives of *o*-HBDI. A schematic of the ESIPT of *o*-HBDI is also shown. For the keto isomer, a representative resonance structure is shown. The red asterisks denote electronic excited states.

To suppress the conformational relaxation leading to low fluorescence quantum yield, an ortho isomer (*o*-HBDI) of the GFP chromophore was synthesized (see Scheme 1), which holds an intramolecular hydrogen bond to form a planar seven-membered ring structure [14]. Upon photoexcitation of *o*-HBDI, ultrafast excited state intramolecular proton transfer (ESIPT) occurs, which results in a change of the hydroxyl group from enol to the keto form [14]. As the length of the π-conjugation system is extended upon ESIPT, the excited state of the keto isomer is stabilized to yield a redshifted fluorescence. Derivatives of *o*-HBDI, shown in Scheme 1, were also synthesized to investigate the effect of electronic properties on the ESIPT reaction [15]. For example, energy of the intramolecular hydrogen bond is affected by the electron donating or withdrawing substituents. The strong electron-withdrawing (NO_2_) substituent in **1** increases acidity of the hydroxyl proton to result in a strong hydrogen bonding, which may affect the energetics and dynamics of the ESIPT and related reactions [16]. In addition, the modification of the photophysical properties results in a wide spectral range of fluorescence [15]. The broad tuning range of emission wavelengths together with the large Stokes shift, which reduces self-absorption, are valuable attributes for photonics applications such as organic light-emitting diodes [17,18,19].

An ESIPT rate, in general, ranges from a few femtoseconds to nanoseconds depending on the shape of the excited state potential energy surface (PES) [20,21,22]. The ESIPT dynamics and the overall proton transfer cycle of *o*-HBDI were studied by transient absorption (TA) and time-resolved fluorescence (TF) experiments [23]. The ESIPT rate of *o*-HBDI in cyclohexane was determined to be faster than 25 fs by TF demonstrating that the ESIPT in *o*-HBDI is practically barrierless. In this work, we performed TF experiments on the derivatives of *o*-HBDI to investigate the effect of electronic properties on the ESIPT reaction. By taking advantage of the high time resolution, we focus on the determination of the precise ESIPT rates and the nuclear wave packet (NWP) motions in the excited product state caused by the ESIPT, which are correlated with the structural change and ESIPT dynamics. The experiments, in conjunction with quantum chemical calculations, suggest a detailed molecular picture of ESIPT dynamics of the ortho-GFP chromophore.

## 2. Results

### 2.1. Steady-State Spectroscopy

**Figure 1:**
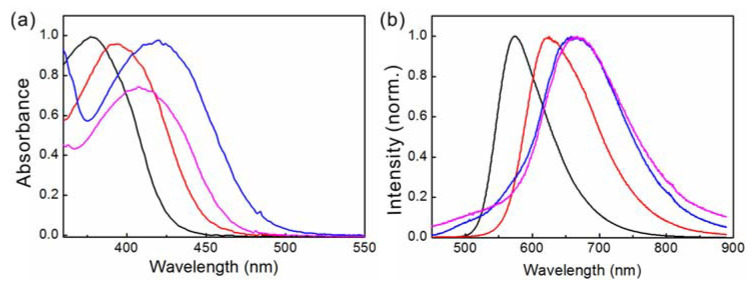
Steady-state (**a**) absorption and (**b**) fluorescence spectra of **1** (black), **2** (red), **3** (blue), and **4** (magenta) in chloroform.

Figure 1 shows the steady-state spectra of the four compounds in chloroform illustrated in Scheme 1. Both the absorption and emission spectra blueshift according to the electron-withdrawing strengths of the substituents in the order of **1** (NO_2_) > **2** (Br) > **4** (H, phenyl) > **3** (OCH_3_). Details of the stationary spectroscopies and time-resolved studies at 1 ps resolution were reported previously [15]. In their quantum chemical calculations, electron densities of the highest occupied molecular orbital (HOMO) and lowest unoccupied molecular orbital) (LUMO) lean more to the phenol and imidazolone moieties, respectively, in the *o*-HBDI derivatives, and the vertical excitations mainly arise from the HOMO-LUMO transition. Therefore, the substitution of the strong electron-withdrawing group at the R_1_ position, which stabilizes the HOMO, causes blueshifts of the absorption and emission spectra. As the phenyl substituent in the imidazolone moiety is weakly electron donating, which pushes the LUMO energy higher, the absorption spectrum of **4** is blueshifted compared to **3**.

All the compounds show large Stokes shifts of over 9100 cm^−1^ except **3,** as listed in Table 1, implying ESIPT. In addition, normal emissions from the enol isomers are absent in the steady-state fluorescence spectra suggesting that the ESIPT is ultrafast. Although it is still much larger than a typical fluorophore in liquid, the Stokes shift of **3** is significantly smaller than those of the other derivatives. In fact, geometry optimization of the excited state by the quantum chemical calculation yields the enol form for **3** (*vide infra*). Therefore, evidence from stationary spectra may suggest that the enol and keto isomers of **3** are in equilibrium in the excited state.

### 2.2. Time-Resolved Fluorescence

**Figure 2:**
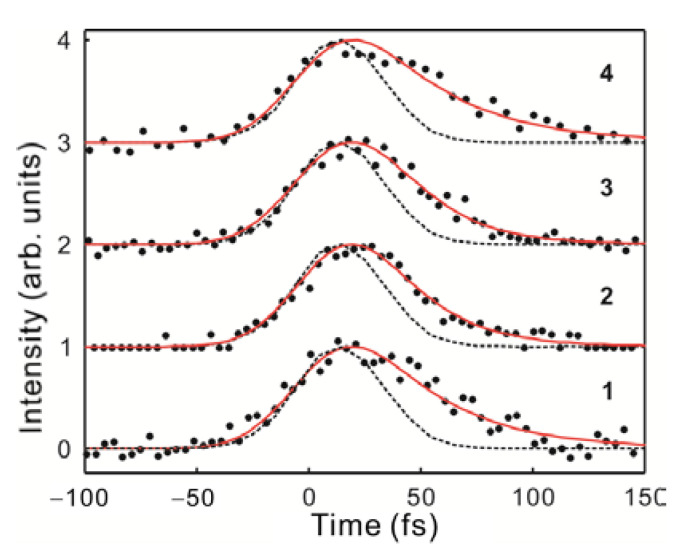
TF signals that were detected at the enol emission wavelengths. The red lines represent single exponential fits, and the dashed lines represent the cross-correlation between the pump and gate pulses.

TF signals that were detected at the enol emission wavelengths are shown in Figure 2. Although hydrogen bond strengths of the four compounds are different, they all show ultrafast decay. The decay rate of the enol emission can be assigned to the ESIPT rate. In particular, because the TFs record spontaneous emission of the enol isomers from S_1_ to S_0_, they solely represent the dynamics of the S_1_ state unlike a TA signal which consists of the ground state contribution, product absorption, as well as stimulated emission. The decay time constants that were obtained from single exponential fits are listed in Table 1, and they are around 30 ± 6 fs, comparable to that of *o*-HBDI that was reported previously [23]. Although the centers of the stationary spectra are shifted according to the electronic properties of the substituents, the Stokes shifts are comparable for all four compounds suggesting that the energetics dictating the ESIPT are similar, giving essentially the same ESIPT rate.

**Figure 3:**
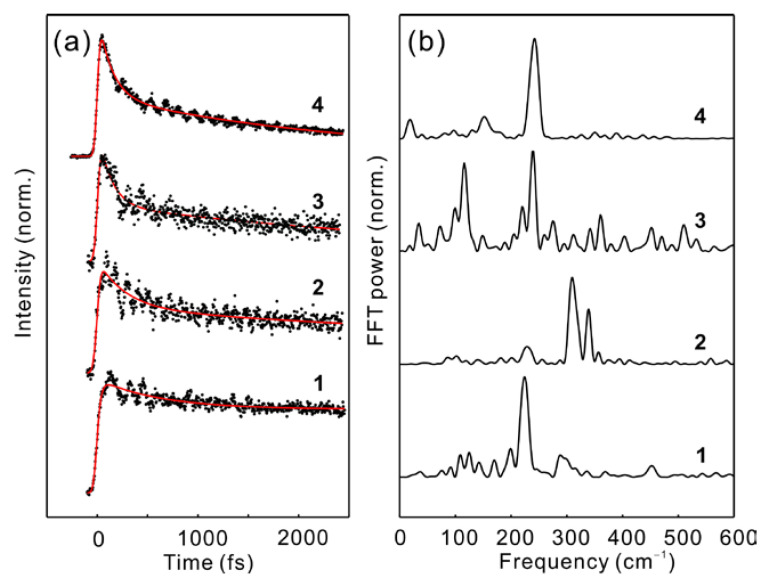
(**a**) TF signals that were detected at the keto emission wavelengths. Residuals of the three exponential fits (red lines) are Fourier transformed to give the spectra in (**b**).

Femtosecond TF signals detected at the keto emission wavelengths are shown in Figure 3. As the modulation of the TF intensity is maximum near the half maximum point of a fluorescence spectrum, we intentionally measured the TF at the blue side of the steady-state fluorescence for the keto isomers. The results of the three-exponential fits consisting of one rise and two decay components are listed in Table 1. The rise component of the keto emission should correspond to the decay component of the enol emission, that is, the ESIPT kinetics. Larger uncertainty in the rise time is due to the large amplitude oscillations in the TF signals arising from the NWP motions of the keto isomer in the excited state. The enol decay and the keto rise times match well for all four compounds. To be more precise, the decay at the enol emission wavelength is the rate of the initial NWPs leaving the Franck–Condon region. Therefore, the agreement of the two time constants indicates that the ESIPT reaction is indeed barrierless and proceeds coherently.

The TF signals at the keto emission exhibit two additional decay components τ_2_ and τ_3_, listed in Table 1. Similar decay kinetics were reported previously on the derivatives of *o*-HBDI, and they were also analyzed in terms of two time constants [15]. The propensity of the decay is evident from Table 1; as the electron-donating character increases, τ_2_ becomes shorter and its amplitude (A_2_) increases, that is, the TF decay becomes faster. The slower time constant (τ_3_) also becomes shorter with increasing electron-donating character. The enol isomers in the ground state have planar geometries because of the hydrogen bonding. The faster decay of TF may indicate a more flexible geometry of the keto isomer with the electron-donating substituent, which in turn suggests weaker hydrogen bonding for the excited keto isomer. Cui et al., by employing a dynamic simulation, proposed that internal conversion (IC) occurs within 330 fs through an S_1_/S_0_ minimum energy conical intersection (CI), which has a nonplanar structure [24]. NWPs that were observed in the excited state together with quantum chemical calculations indicate that **3** may undergo large structural relaxation following the ESIPT, in line with this notion (*vide infra*).

In addition to the decay, large amplitude oscillations of the TF signals arising from the NWP motions in the excited state are apparent. As the ESIPT rates are fast enough to excite the reaction products impulsively, the oscillations have structural and dynamical information of the excited product state. A Fourier transform of the residual that was obtained from the three-exponential fit gives the coherent vibrational spectrum (CVS) that was attained via TF (CVSF), shown in Figure 3b. The CVSFs show vibrational peaks of frequencies below 600 cm^−1^ because of the finite time resolution of the TF apparatus. An oscillation of frequency ω is attenuated by a factor of exp(−σ^2^ω^2^/2), where σ is the standard deviation of the instrument response function (IRF) assuming a Gaussian function. Therefore, a 500 cm^−1^ mode would be attenuated by a factor of 3.6 due to the finite 40 fs time resolution of this work. We also exploited the linear prediction singular value decomposition (LPSVD) method to retrieve the CVSFs [25,26], and the major peaks that were acquired by LPSVD are listed in Table 2. The LPSVD method is appropriate for low signal-to-noise ratio data and a complex time trace that consists of multiple exponential and damped sinusoidal functions [27]. Within the Condon approximation, the amplitude of an NWP is proportional to the vibrational reorganization energy [28], which can be attained theoretically from quantum chemical calculations. Although the Fourier transform gives an unbiased CVSF, it is not straightforward to retrieve amplitudes of oscillations at time zero because the amplitude depends on the height and width of the peaks, whereas LPSVD analysis gives the amplitudes directly. For example, the 291 cm^−1^ peak of **1** looks small in the Fourier power spectrum, but it is one of the major peaks in the CVSF by LPSVD. Moreover, subtraction of the exponential components by nonlinear least square fits may introduce artifacts and uncertainty.

### 2.3. Quantum Chemical Calculations of CVSF

Quantum chemical calculations of the ground and excited states were performed by the density functional theory (DFT) and time-dependent DFT (TDDFT), respectively, at the B3LYP/6-311++G(d,p) level using the Gaussian 16 software package [29]. C_S_ symmetry was imposed for the geometry optimization. The optimized geometries of the ground and excited states are enol and keto forms, respectively, for all the compounds except **3**, whose optimized excited state geometry is the enol form as shown in Table 3. Harmonic frequency scale factor was not used because it is close to 1 for the quantum calculation method that was employed in this work [30].

If a reaction is impulsive, the amplitude of an NWP in the product state is proportional to the vibrational reorganization energy, which is proportional to the square of the vibrational displacement between the PES of the reactant and product [31]. We have calculated the CVSFs of the products in the excited state by assuming that the keto isomers are generated instantaneously after photoexcitation. We expect that this approach is valid for the vibrational modes with periods that are longer than roughly twice the ESIPT time, that is, modes below ~600 cm^−1^. We used quantum chemical calculations to obtain the geometrical displacement between the enol isomer in the ground state and the keto isomer in the excited state [32]. Projecting the geometrical displacement onto the normal modes of the keto isomer gives the vibrational displacements, vibrational reorganization energies, and a CVSF. Peaks of the CVSFs that were calculated in this way were scaled by exp(−σ^2^ω^2^/2) to account for the finite time resolution of the TF experiment.

**Figure 4:**
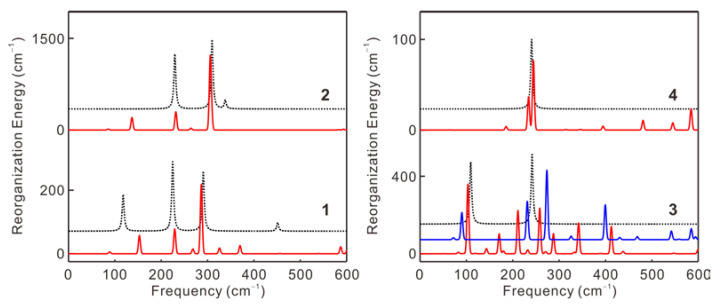
CVSFs that were obtained by LPSVD (black dashed lines) are compared with the calculated CVSFs (red solid lines). The blue solid line represents the calculated CVSF with the excited state geometry forced to be the keto isomer. The widths of the peaks in the experimental and calculated CVSFs are set to 5 cm^−1^ arbitrarily. The *y*-scales correspond to the calculated CVSFs (red lines); note that they are different.

The calculated CVSFs are shown in Figure 4, and they were compared with the experimental CVSFs that were attained by the LPSVD analysis. The agreement between the theory and experiment is excellent for **1**, **2**, and **4** in terms of both frequencies and amplitudes. As we cannot obtain the stable enol isomer in the excited state for **1**, **2**, and **4**, we cannot calculate the CVSFs of the enol isomer. For illustration purposes, we forced **2** to be in the enol form in the excited state, calculated the CVSF, and compared with the experiment, which is shown in Figure S1 in the Supporting Information. Several important conclusions can be drawn from the excellent agreement: (1) the product is indeed the excited keto isomer. The CVSF is the vibrational spectrum of the product with amplitude information, and it can be a powerful technique to identify the reaction product as demonstrated previously [31]; (2) the ESIPT is ultrafast and occurs impulsively for the vibrations below 600 cm^−1^; and (3) it suggests that minimal structural relaxation is involved following the ESIPT retaining the hydrogen bond in the excited keto isomer at least for the time window of the experiment, which is a few picoseconds. Structural relaxation may lead to attenuation of the vibrations coupled strongly with the relaxation coordinates [33], which results in a disparity between the experiment and calculated CVSFs in this way.

The calculated and experimental CVSFs of **3** barely match in terms of both frequencies and amplitudes. The discrepancy can be implied by the quantum chemical calculation, which shows that the optimized structure of **3** in the excited state is the enol isomer (Table 3). The match is not good when the excited state geometry is forced to be the keto isomer. Equilibrium between the enol and keto isomers is also not likely as the experimental CVSF does not match with a sum of the calculated CVSFs of the enol and keto isomers. Therefore, occurrence of the ESIPT may not be clear for **3**. However, we conclude that the ESIPT does occur for **3** based on the following observations: (i) the calculated fluorescence wavelength (Table 3) constrained to the keto form matches well with the experiment; (ii) the Stokes shift is still large; and (iii) the decay and rise times of the TFs at the enol and keto emission wavelengths match well. Then, the mismatch in the CVSFs suggest that there can be an extensive structural relaxation following ESIPT in **3**, which in turn suggests that the hydrogen bonding in the excited state is weak because of the electron donating methoxy group.

## 3. Discussion

The fast (τ_2_) and slow (τ_3_) time constants in the TF signals of the keto isomer are assigned to the IC following ESIPT. Litvinenko et al. reported the population dynamics independent of the orientational relaxation using ultrafast polarization spectroscopy for *p*-HBDI in solution [10]. The IC rate showed strong temperature dependence and weak coupling to the viscosity, which led to the conclusion that the IC is promoted by the volume-conserving motion. Mandal et al. reported TFs of *p*-HBDI in neutral and anion forms [11]. The TFs consist of two exponentials that were independent of the detection wavelengths. The neutral state decays faster than the anionic state regardless of the viscosity and polarity of solvents. The excited state relaxation dynamics were discussed in terms of a two-state two-mode model that involves intramolecular vibrational redistribution followed by IC. In contrast, the IC of *o*-HBDI showed solvent viscosity dependence [23]. Theoretical investigation of *o*-HBDI by Cui et al. employing various computational methods and a semiempirical surface-hopping dynamics simulation showed that the IC occurs through an S_1_/S_0_ minimum energy CI which has a nonplanar structure [24]. Using the simulation, they showed that the IC occurs through this CI channel within 330 fs, and there is no evidence of cis-trans isomerization. The main structural difference between the S_1_ minimum and the CI is the rotation of the single C-C bond that connects the phenyl ring and the imidazolone ring. Therefore, it was argued that weakening of the hydrogen bond causes rotational fluctuation of the single bond leading to the conical intersection and the internal conversion. Our femtosecond and picosecond TF data show faster IC with increasing electron-donating properties. This observation suggests that a weaker hydrogen bond by the electron-donating group makes the molecule flexible to undergo structural deformation and IC through the CI. NWPs that were observed in the excited state together with quantum chemical calculations indicate that **3** may undergo large structural relaxation following the ESIPT, which is consistent with the previous report.

**Figure 5:**
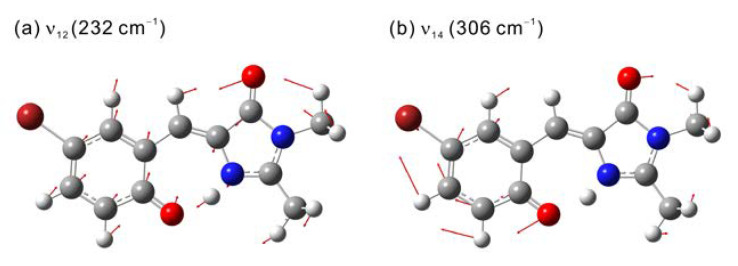
Normal modes (**a**) ν_12_ and (**b**) ν_14_ of **2** in the excited keto isomer showing large vibrational reorganization energies with respect to the ESIPT. The red arrows indicate displacement vectors.

Analogous to the typical ESIPT molecules showing ultrafast ESIPT such as 2-(2′-hydroxyphenyl)benzothiazole (HBT) and 10-hydroxybenzo[H]quinoline (HBQ), the vibrational modes excited strongly in the keto isomers (strong peaks in the CVSFs) involve large displacements of atoms around the hydrogen bond. In particular, the distance between the (O, N) atoms is modulated strongly, which leads to the model of skeletal mode-assisted ESIPT [28,34,35,36]. In this model, the vibrational modes with large vibrational displacements for the ESIPT involve large amplitude in-plane motion to make the distance between the donor O and acceptor N atoms close to making the PES for ESIPT barrierless. The two peaks appearing strongly in the CVSFs of **1** (ν_13_, ν_15_) and **2** (ν_12_, ν_14_) are actually very similar vibrational modes, and they also modulate the O--N distance as shown in Figure 5. Therefore, it is tempting to argue for the skeletal mode-assisted ESIPT. However, the NWPs that were observed in the TF are the result of the ESIPT, and they do not drive the ESIPT. The fact that the calculated CVSFs assuming impulsive ESIPT predict the experimental CVSFs accurately supports this conclusion. In this model, amplitudes of the NWPs will depend strongly on the ESIPT reaction rate because phases of the vibrations whose periods are longer than ESIPT time will be randomized by the ESIPT process. Experimental measurements of the dependence of the NWP amplitudes on the ESIPT rate will be an interesting topic to verify this model.

## 4. Materials and Methods

Femtosecond TF was acquired by the fluorescence upconversion method utilizing noncollinear sum frequency generation (SFG) described elsewhere [37]. The light source was based on a home-built cavity dumped Ti:sapphire laser pumped by a frequency-doubled Nd:YVO_4_ laser (Verdi, Coherent Inc., Santa Clara, CA, USA). A pump pulse around 400 nm was generated by the second harmonic generation (SHG) in a 100 µm thick β-barium borate (BBO) crystal. The pump pulses were attenuated to less than 1 nJ to avoid photodamage of the samples. The residual fundamental laser pulses around 800 nm were used as gate pulses for the SFG. Group velocity dispersion (GVD) was compensated carefully by prism pairs and negative dispersion mirror pairs (LAYERTEC GmbH, Mellingen, Germany). The effects of group velocity mismatch (GVM) and phase front mismatch (PFM) were minimized by optimizing the crossing angle between the fluorescence and the gate beams [37]. The IRF that was determined by the cross-correlation between the scattered pump and the gate pulses was around 40 fs full width at half maximum (FWHM) when a 100 µm thick BBO was used.

Picosecond TF was achieved by the time-correlated single photon counting (TCSPC) method using an apparatus that was reported previously [38]. The IRF of the TCSPC apparatus had a width (FWHM) of 50 ps.

The samples were synthesized as reported previously [15]. The ^1^H and ^13^C NMR spectra of **1**–**4** are shown in the Supporting Information Figures S2–S9. The sample solutions were flown in a 100 µm thick flow-cell to minimize photodamage.

## 5. Conclusions

In this work, we investigated the excited state dynamics of the ortho-hydroxy isomer (*o*-HBDI) of the GFP chromophore by TF. The time-resolution of the TF apparatus was high enough to allow recording of the nuclear wave packets in the excited product state as well as the dynamics of the photoexcited state. In particular, derivatives of *o*-HBDI that have substituents were prepared to investigate the effect of the electronic properties on the excited state dynamics. All the compounds that were employed in this work showed ultrafast ESIPT occurring in about 30 fs, except for a compound that was substituted by an electron-donating methoxy group, in which the occurrence of the ESIPT is inconclusive. Although the ESIPT rates were not affected by the electronic properties of the substituents suggesting barrierless reaction, subsequent relaxation dynamics following ESIPT are distinct. When an electron-withdrawing group is substituted, the hydrogen bond is strengthened keeping the molecule planar rigid structure, which slows the internal conversion to the ground state.

The TF signals at the keto emission wavelengths display oscillations due to the wave packet motions of the product keto isomers. We performed quantum chemical calculations to reproduce the CVSFs, assuming impulsive transition from the enol isomer in the ground state to the keto isomer in the excited state. The excellent agreement of the experimental and calculated CVSFs corroborates the ultrafast ESIPT. Prominent peaks in the CVSFs can be assigned to the vibrational modes that involve large displacements of the atoms in the seven-membered chelate ring that is formed by the hydrogen bond. Moreover, they are the same vibrational modes in different derivatives. Together with the excellent agreement of the experimental and calculated CVSFs, this observation suggests that the vibrational modes in the product keto isomer are excited by the ESIPT reaction acting as the impulsive excitation of vibrations. In this case, the ESIPT rate should control the amplitudes and phases of the wave packets in the product state. Currently, we are verifying this view by recording the CVSFs for a system where the ESIPT rates can be varied systematically.

## Figures and Tables

**Table 1:** Stokes shifts, average lifetimes, and the exponential fit results of the TF signals detected at the enol and keto emission wavelengths.

Compounds	Stokes Shift (cm^−1^)	τ_avg._ (ps)	λ_det_ (nm)	A_1_	τ_1_ (fs)	A_2_	τ_2_ (fs)	A_3_	τ_3_ (ps)
**1**	9100	480 ^a^	430	1.0	36				
			550	−1.0	37	0.24	235	0.76	15
**2**	9380	10 ^a^	440	1.0	24				
			580	−1.0	37	0.47	236	0.53	7.9
**3**	8560	2 ^b^	450	1.0	24				
			630	−1.0	23	0.61	105	0.39	5.1
**4**	9370	1 ^b^	450	1.0	38				
			630	−1.0	16	0.62	131	0.38	2.4

^a^ data by TCSPC. ^b^ data by fluorescence upconversion experiment.

**Table 2:** Major peaks of the CVSFs at the keto emission wavelengths and their assignments.

Compounds	Amplitude	Freq. (cm^−1^)	Dephasing Time (fs)	Vib. Mode	Cal Freq. (cm^−1^)
**1**	0.54	118	520	ν_10_	153(?)
	1.0	225	690	ν_13_	229
	0.88	291	360	ν_15_	287
	0.12	451	2500	ν_21_	456
**2**	0.78	229	510	ν_12_	232
	1.0	310	1100	ν_14_	306
	0.12	338	3200	ν_17_	338
**3**	0.9	109	530	ν_6_	103
	1.0	242	370	ν_13_	232
**4**	1.0	241	1500	ν_12_	245

**Table 3:** Bond lengths (Å) around the hydrogen bond moiety and fluorescence wavelengths of the S_1_ state calculated by the TDDFT method with B3LYP/6-311++G(d,p).

Compound	λ (nm)	*d* (O-H)	*d* (N-H)	*d* (O-N)
**1**	574 (0.171)	1.6606	1.0415	2.6106
**2**	595 (0.155)	1.5138	1.0776	2.5381
**3**	581 (0.090)	1.0728	1.4438	2.5042
**3** *^a^*	669 (0.151)	1.6281	1.0000	2.5622
**4**	672 (0.185)	1.6206	1.0570	2.5999

*^a^* The N-H bond length was fixed to 1.0 Å during the geometry optimization. Energy is 1200 cm^−1^ higher for the constrained geometry.

## Data Availability

The data presented are contained within the article, and raw data available upon request.

## Supplementary Materials

The supporting information can be downloaded at: https://www.mdpi.com/article/10.3390/ijms24043448/s1.
