# Supplementary material for: Excited State Intramolecular Proton Transfer Dynamics of Derivatives of the Green Fluorescent Protein Chromophore
Source: Int J Mol Sci. 2023 Feb 9;24(4):3448. doi: 10.3390/ijms24043448 (PMC9962057; doi:10.3390/ijms24043448)
Supplement: Supplementary file 1 [file ijms-24-03448-s001.zip › ijms-2188871-supplementary.pdf]

Supporting Information

Excited State Intramolecular Proton Transfer Dynamics of Derivatives of the Green Fluorescent Protein Chromophore

Junghwa Lee [1,†,‡], Pyoungsik Shin [1,†], Pi-Tai Chou [2,*] and Taiha Joo [1,*]

[1] Department of Chemistry, Pohang University of Science and Technology (POSTECH), Pohang 37673, Republic of Korea

[2] Department of Chemistry, National Taiwan University, Taipei 10617, Taiwan, China

* Correspondence: chop@ntu.edu.tw (P.-T.C.); thjoo@postech.ac.kr (T.J.)

† These authors contributed equally to this work.

‡ Current Address: Samsung Electro-Mechanics Co., Suwon 16674, Republic of Korea

Contents

Figure S1. Experimental and calculated CVSFs of **2** in the keto and enol forms.

Figure S2. ^{1}H NMR spectrum of **1**.

Figure S3. ^{13}C NMR spectrum of **1**.

Figure S4. ^{1}H NMR spectrum of **2**.

Figure S5. ^{13}C NMR spectrum of **2**.

Figure S6. ^{1}H NMR spectrum of **3**.

Figure S7. ^{13}C NMR spectrum of **3**.

Figure S8. ^{1}H NMR spectrum of **4**.

Figure S9. ^{13}C NMR spectrum of **4**.

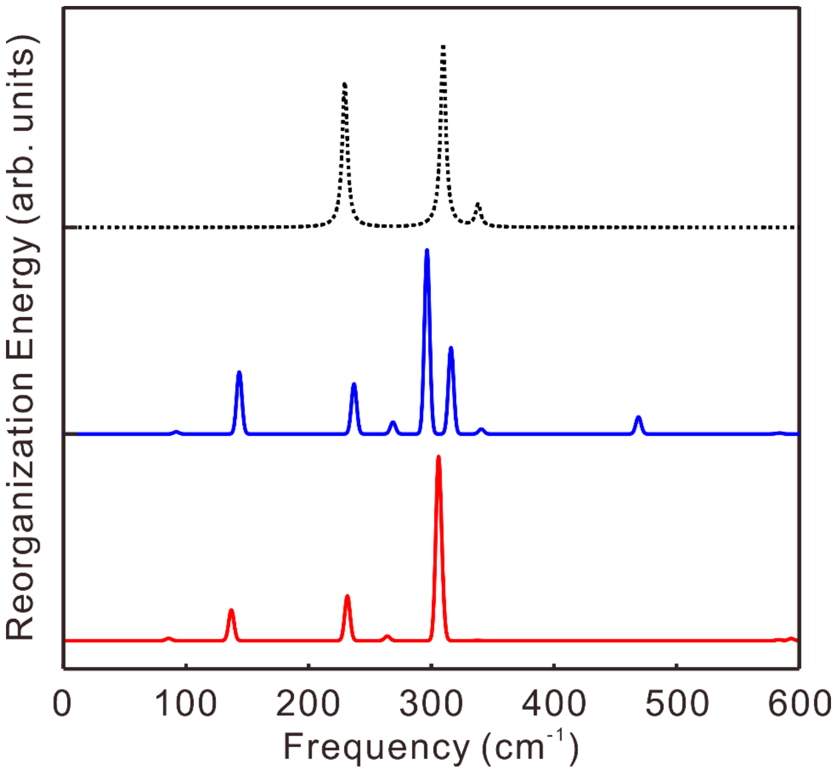

Figure S1. Experimental CVSF of **2** obtained by LPSVD (black dashed line) is compared with the calculated CVSFs of the keto (red) and enol (blue) isomers in the excited state. Because the keto isomer is the only species stable in the excited state, the OH bond length was fixed to that of the enol isomer in the ground state. The widths of the peaks in the experimental and calculated CVSFs are set to 5 cm^{-1} arbitrarily.

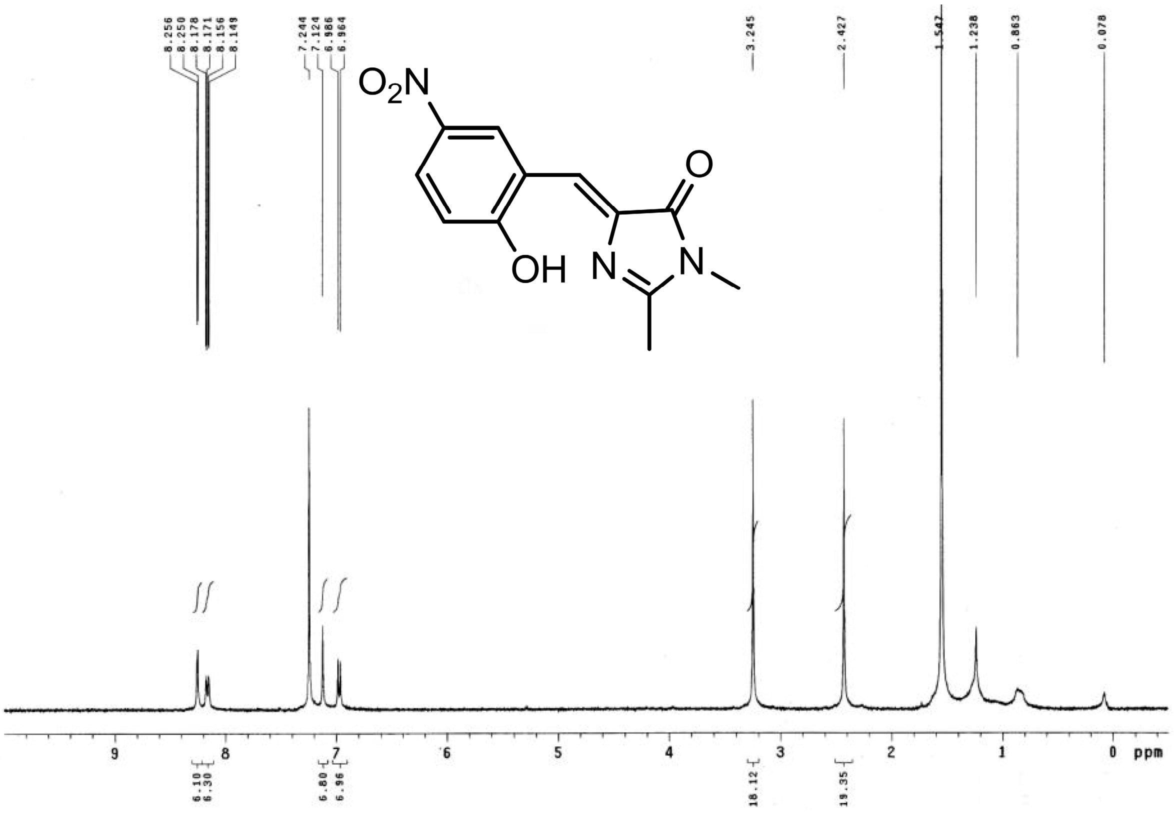

Figure S2. ^{1}H NMR of **1**.

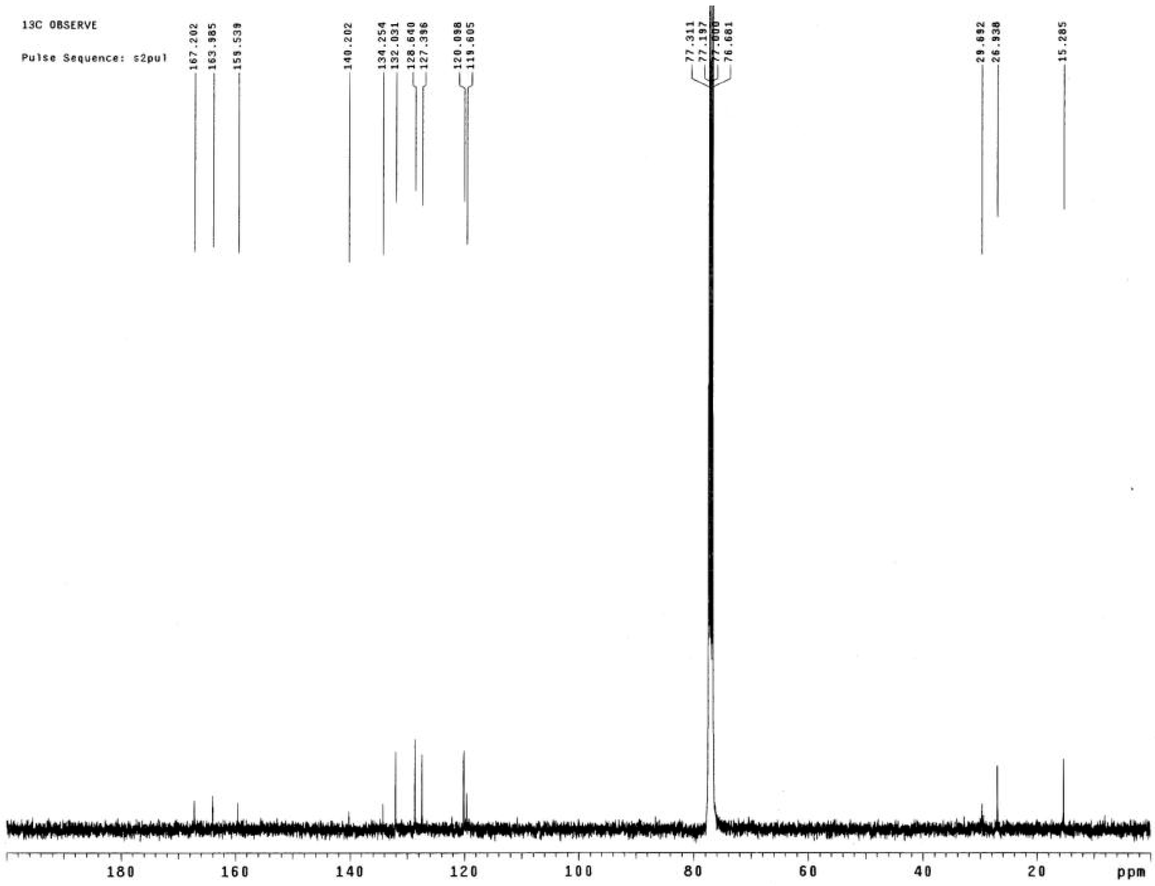

Figure S3. ^{13}C NMR of **1**.

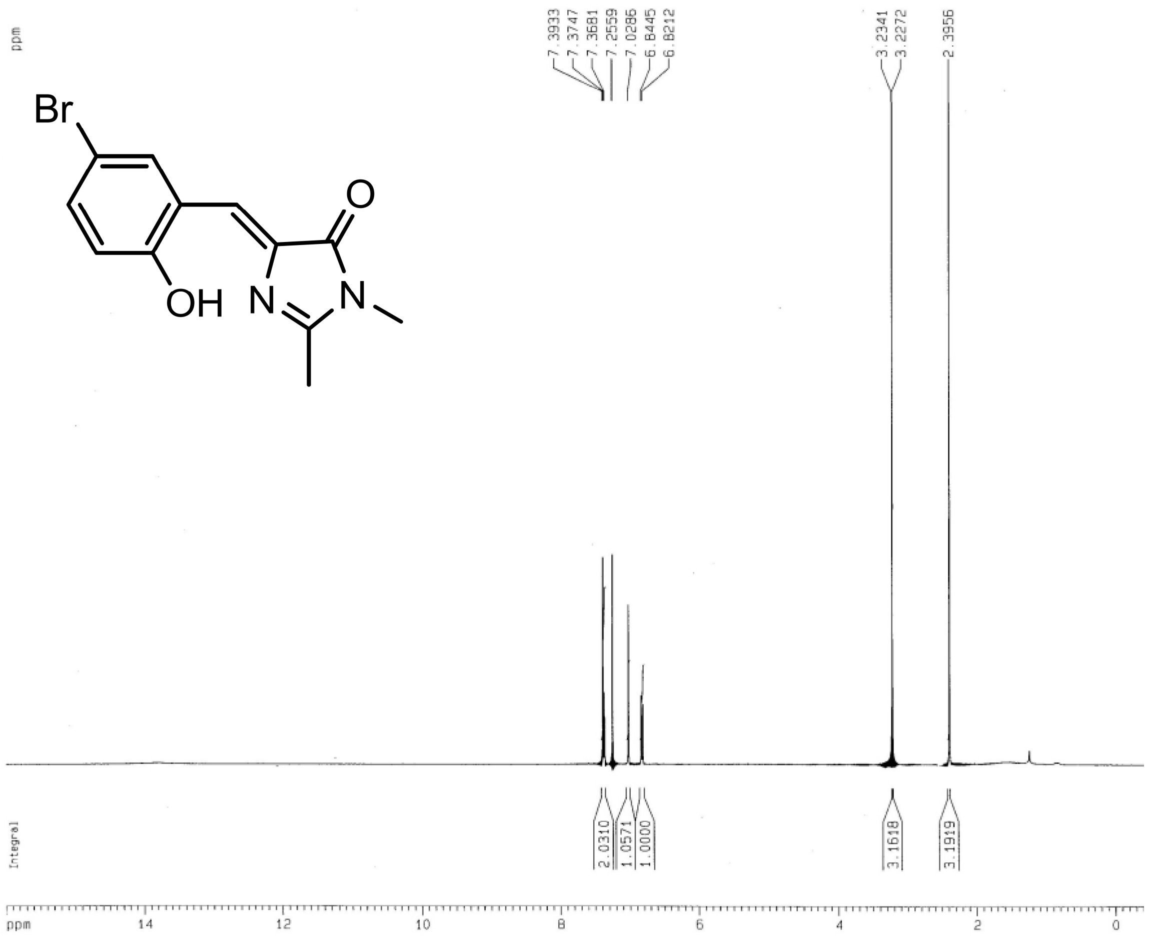

Figure S4. ^{1}H NMR of **2**.

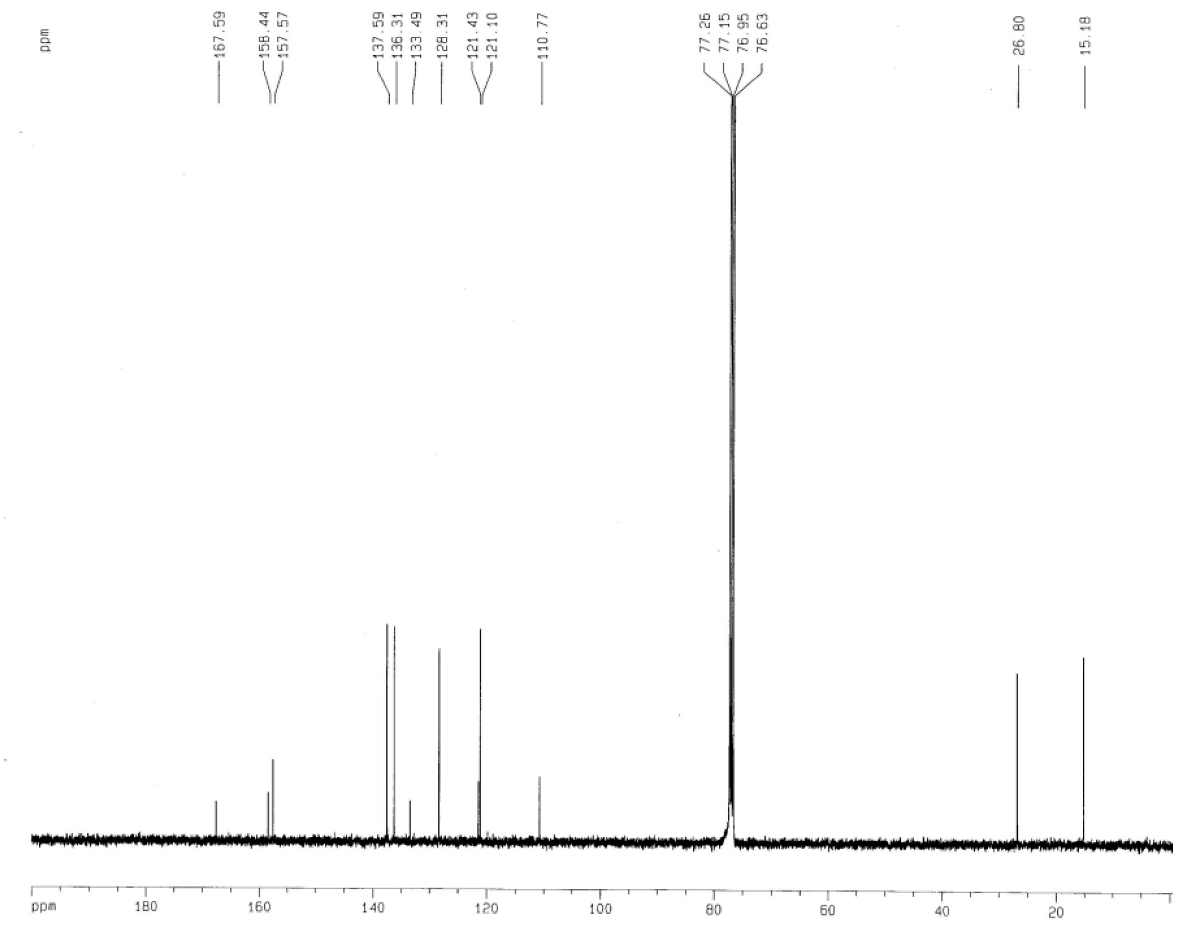

Figure S5. ^{13}C NMR of **2**.

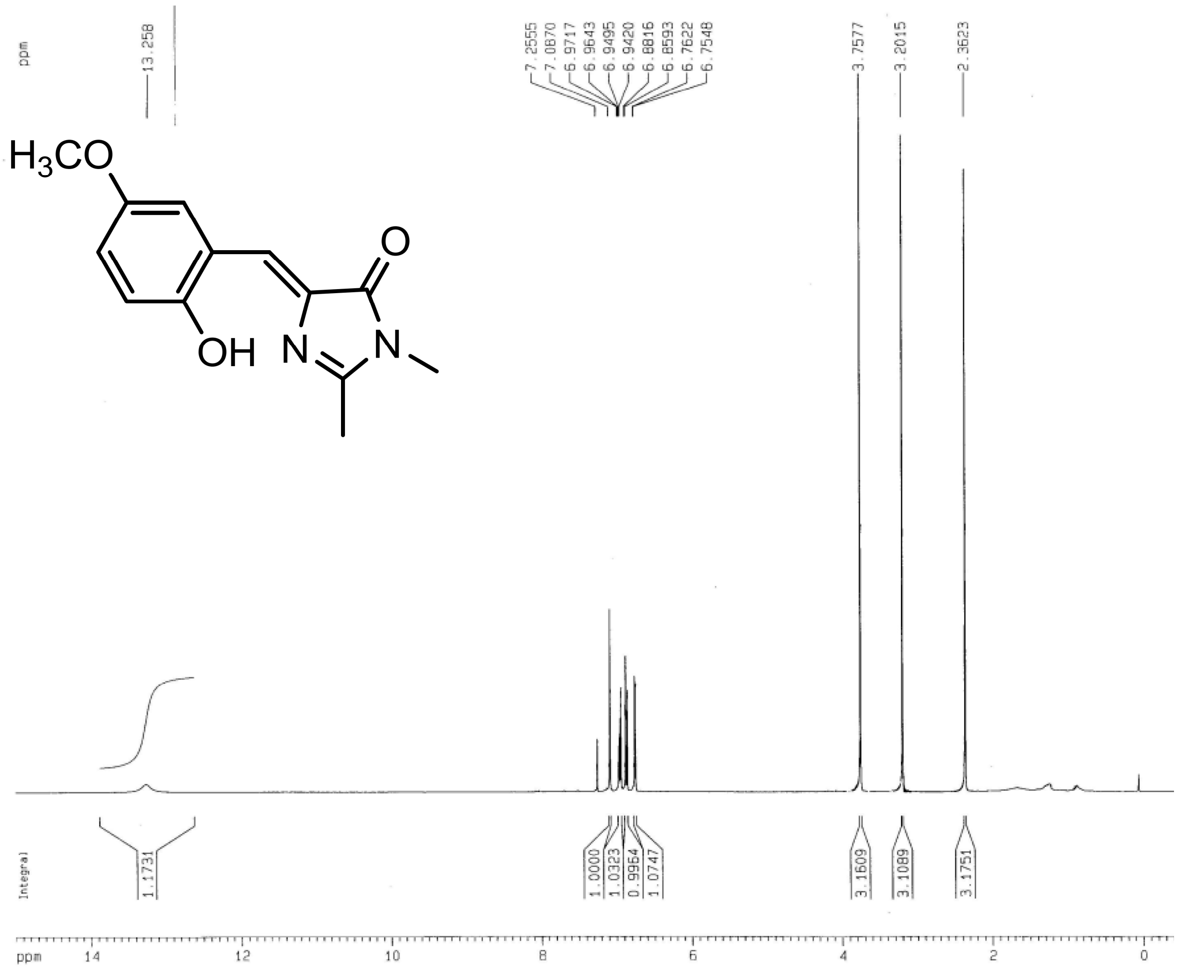

Figure S6. ^{1}H NMR of **3**.

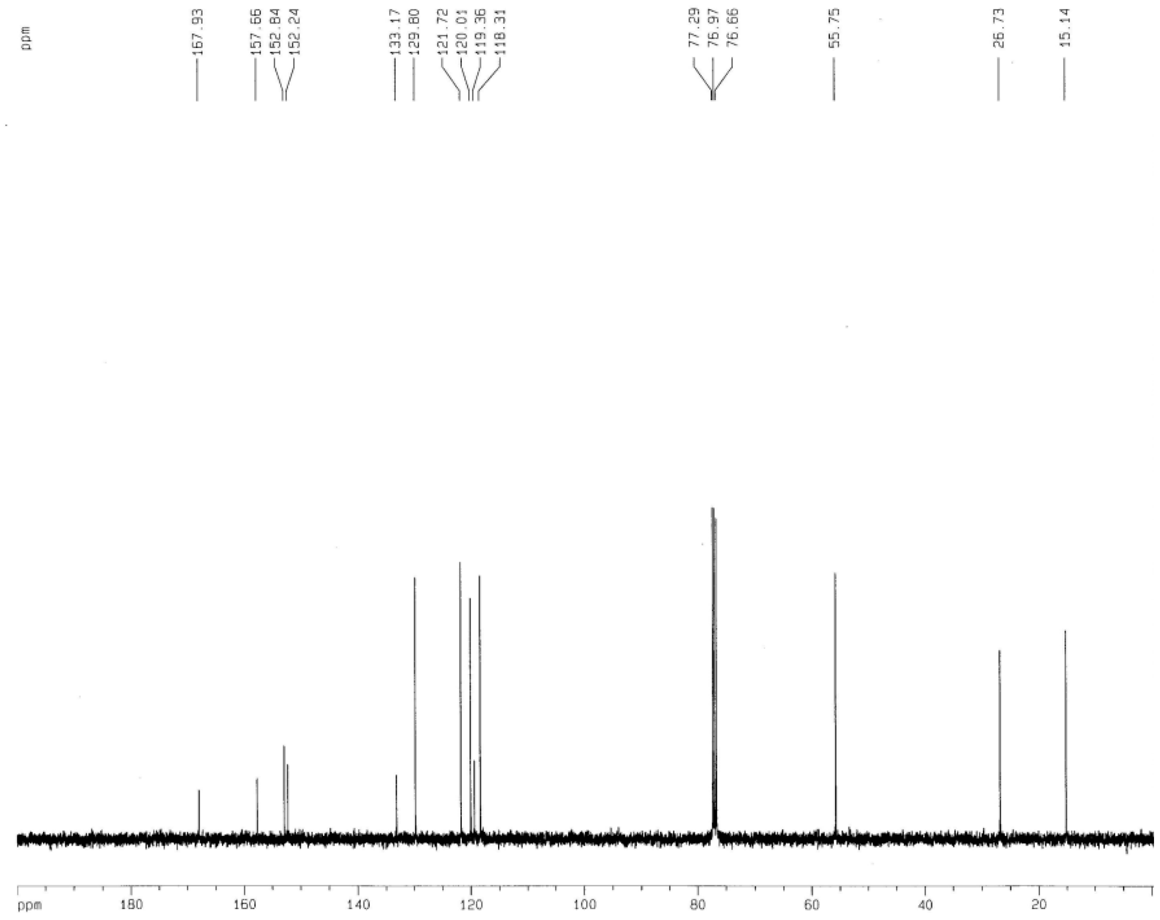

Figure S7. ^{13}C NMR of **3**.

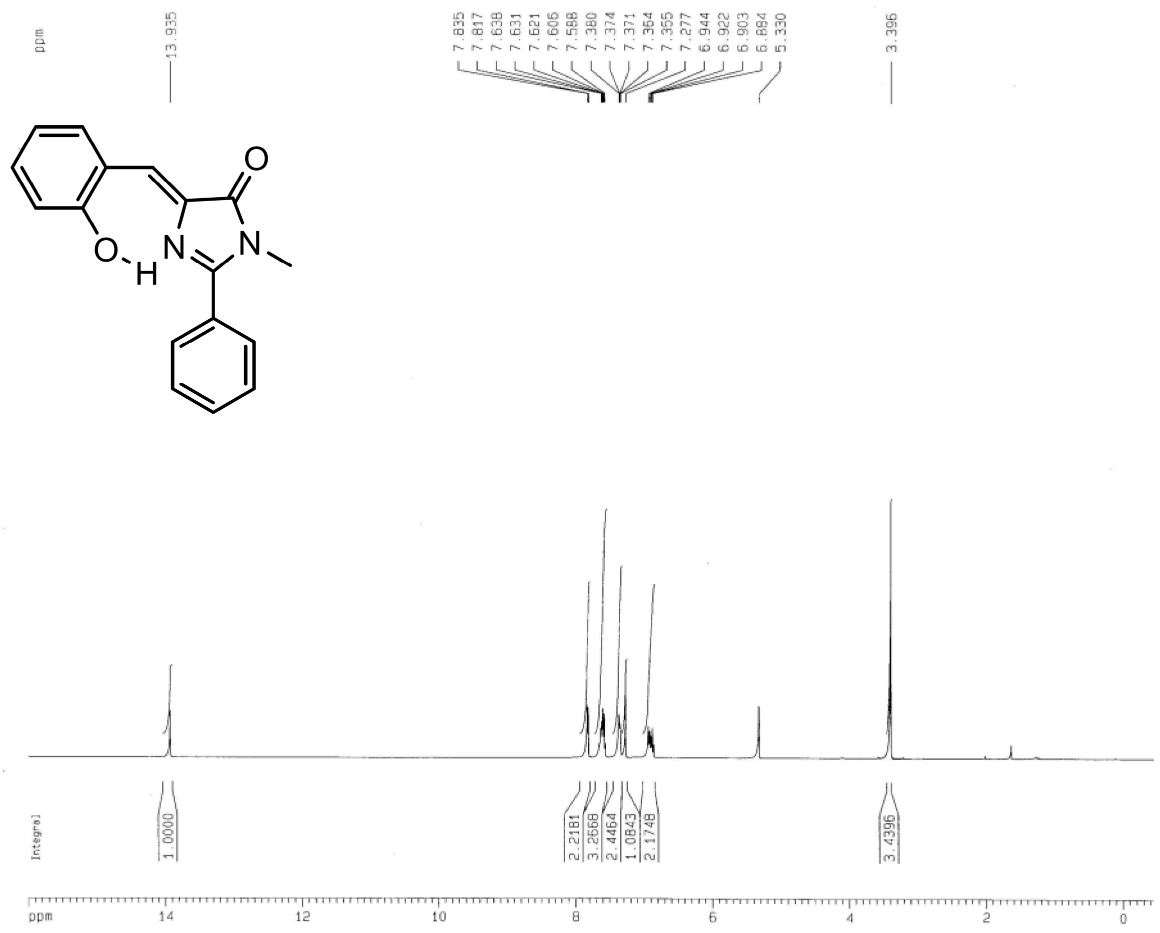

Figure S8. ^{1}H NMR of **4**.

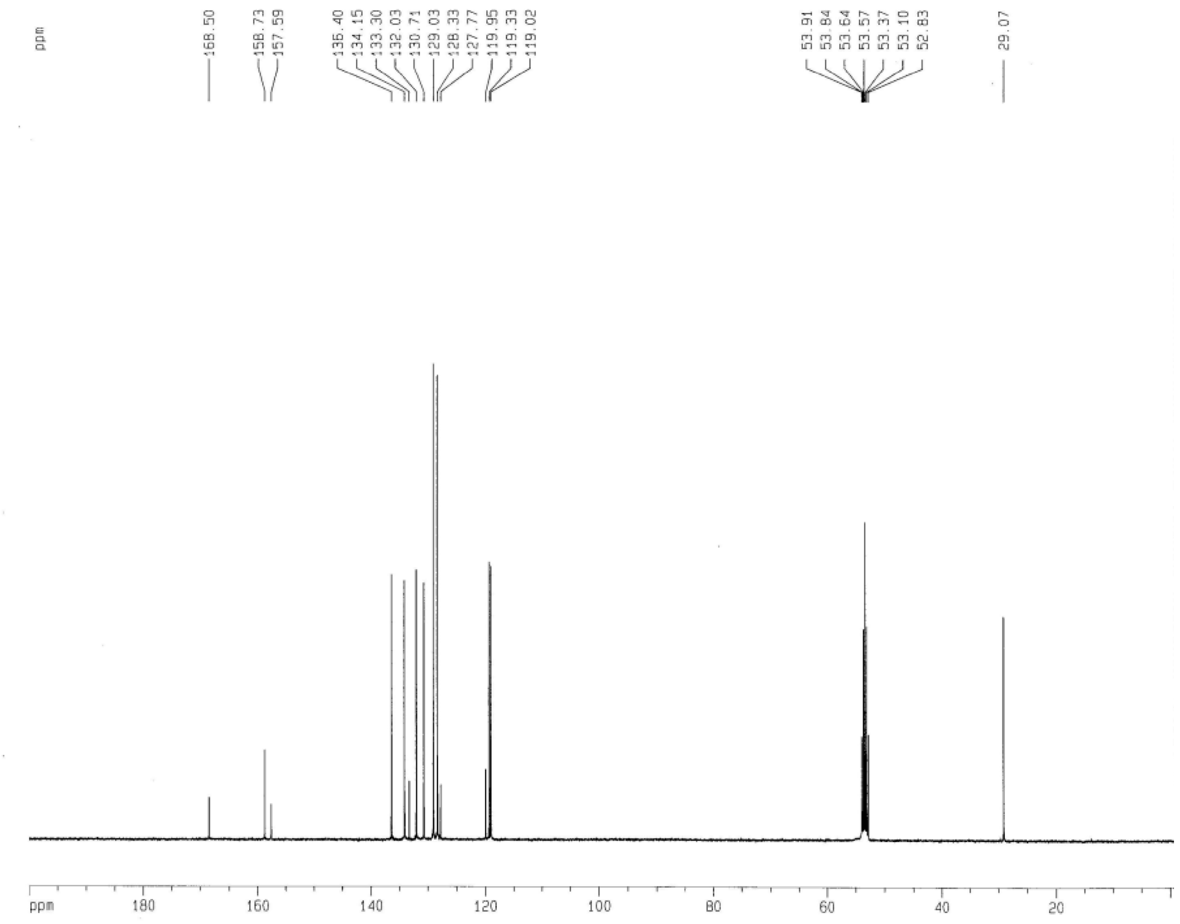

Figure S9. ^{13}C NMR of **4**.
